# Prognostic value of vasodilator stress perfusion cardiovascular magnetic resonance after inconclusive stress testing

**DOI:** 10.1186/s12968-021-00785-6

**Published:** 2021-07-05

**Authors:** Théo Pezel, Thierry Unterseeh, Philippe Garot, Thomas Hovasse, Marine Kinnel, Stéphane Champagne, Solenn Toupin, Francesca Sanguineti, Jérôme Garot

**Affiliations:** 1 Institut Cardiovasculaire Paris Sud, Cardiovascular Magnetic Resonance Laboratory, Hôpital Privé Jacques Cartier, Ramsay Santé, 6 Avenue du Noyer Lambert, 91300 Massy, France; 2 Division of Cardiology, Johns Hopkins University, Baltimore, MD 21287-0409 USA; 3 Siemens Healthcare France, 93200 Saint-Denis, France

**Keywords:** Cardiovascular magnetic resonance, Stress testing, Inconclusive stress test, Cardiovascular events, Dipyridamole, Revascularization

## Abstract

**Background:**

While current guidelines recommend noninvasive testing to detect coronary artery disease, stress tests are deemed inconclusive in a quarter of cases. The strategy for risk stratification after inconclusive stress testing is not well standardized. To assess the prognostic value of vasodilator stress cardiovascular magnetic resonance (CMR) parameters and CMR-based coronary revascularization in patients after inconclusive stress testing.

**Methods:**

Between 2008 and 2020, consecutive patients with a first non-CMR inconclusive stress test referred for vasodilator stress perfusion CMR were followed for the occurrence of major adverse cardiovascular events (MACE), defined by cardiovascular death or nonfatal myocardial infarction. CMR-related coronary revascularization was defined as any revascularisation occurring within 90 days after CMR. Univariable and multivariable Cox regressions were performed to determine the prognostic value of each parameter.

**Results:**

Of 1563 patients who completed the CMR protocol, 1402 patients (66.7% male, 69.5 ± 11.0 years) completed the follow-up (median [interquartile range], 6.5 [5.6–7.5] years); 197 experienced a MACE (14.1%). Vasodilator stress CMR was well tolerated without severe adverse events. Using Kaplan–Meier analysis, inducible ischemia and late gadolinium enhancement (LGE) were significantly associated with the occurrence of MACE (hazard ratio, HR: 2.88 [95% CI 2.18–3.81]; and HR: 1.46 [95% CI 1.16–1.89], both p < 0.001; respectively). In multivariable Cox regression, the presence and extent of inducible ischemia were independent predictors of a higher incidence of MACE (HR: 2.53 [95% CI 1.89–3.40]; and HR: 1.58 [95% CI 1.47–1.71]; both p < 0.001; respectively). After adjustment, the extent of inducible ischemia showed the best improvement in model discrimination above traditional risk factors (C-statistic 0.75 [95% CI 0.69–0.81] with C-statistic improvement: 0.12). The study suggested no benefit of CMR-related coronary revascularization in reducing MACE.

**Conclusions:**

In patients with a first non-CMR inconclusive stress test, vasodilator stress CMR has good prognostic value to predict MACE offering an incremental prognostic value over traditional risk factors.

**Supplementary Information:**

The online version contains supplementary material available at 10.1186/s12968-021-00785-6.

## Introduction

Cardiovascular disease (CVD) mortality due to coronary artery disease (CAD) has recently increased, and CAD represents more than $500 million in annual health care costs in the United States alone [1]. While current guidelines recommend a non-invasive stress testing or coronary computed tomography angiography (CTA) for the initial diagnostic management of patients with angina and suspected CAD (class IA) [2, 3], stress tests are deemed inconclusive in up to 15% to 29% of cases [4, 5]. The management of patients with inconclusive stress test is not well standardized and studies reported that < 25% of patients with inconclusive stress test underwent an additional stress test in clinical practice [4, 6]. Moreover, it has been shown that inconclusive stress testing leads to a 140% increase in medical costs at 2 years and a worse prognosis compared to patients with conclusive negative tests [6, 7]. Although some reports support that further testing after first inconclusive stress test may improve diagnostic accuracy of obstructive CAD and risk stratification [6], the management of such patients remain controversial because data are scarce [7]. Vasodilator stress cardiovascular magnetic resonance (CMR) is recognized as an accurate technique to depict inducible myocardial ischemia and infarction with high sensitivity and specificity [8, 9]. A first-line stress CMR-based strategy was recently shown to be non-inferior in terms of outcomes compared to an invasive approach with fractional flow reserve in patients with stable angina [10]. Although several large studies have shown the prognostic value of stress CMR [11, 12], no studies have specifically assessed the prognostic value of stress CMR in targeted patients with a first inconclusive stress test.

The aim of this study was to assess the prognostic value of stress CMR parameters and CMR-based coronary revascularization in consecutive patients referred for stress CMR after a first inconclusive noninvasive stress test.

## Methods

### Study population

Between December 2008 and January 2020, we conducted a single-centre longitudinal study with retrospective enrollment of consecutive patients with a first non-CMR inconclusive noninvasive stress test as the main indication for vasodilator stress perfusion CMR. Inconclusive stress test was defined by exercise electrocardiogram (ECG) or stress echocardiography or single photon emission computed tomography (SPECT) without positive or negative conclusion regarding the diagnosis of CAD [6, 13]. Two expert physicians reviewed the first stress test, using the definitions of positive or negative tests presented in Additional file 1, in accordance with previous studies [6]. Patients without angina or dyspnea on exertion underwent the first stress test during the work-up of known CAD, or because of relatively high CVD risk defined by the presence of at least 2 CVD risk factors (age > 50 years for men or > 60 years for women, diabetes, hypertension, smoking, dyslipidemia, family history of CAD and obesity defined by body mass index (BMI) ≥ 30 kg/m^2^). Exclusion criteria were: (i) contraindication to CMR (cerebral clips, metallic eye implant); (ii) contraindication to dipyridamole (severe asthma or chronic obstructive pulmonary disease, second- or third-degree atrioventricular block); (iii) known cardiomyopathy (e.g. hypertrophic, dilated, or infiltrative) and acute or chronic myocarditis; (iv) known allergy to gadolinium-based contrast medium; and (v) glomerular filtration rate < 30 ml/min/1.73 m^2^. Clinical data were collected according to medical history and clinical examination on the day of stress CMR. All patients gave informed written consent for clinical CMR examination and enrolment in the clinical research study at baseline. The study was approved by the local ethic committee of our institution and conducted in accordance with the Declaration of Helsinki. This study followed the STROBE reporting guidelines for cohort studies.

### Patients follow-up and clinical outcome

The follow-up consisted of a clinical visit as part of usual care (72%) or by direct contact with the patient or the referring cardiologist (28%). Data collection was ended on June 2020. Cardiovascular events were checked by medical reports collected from the corresponding hospitals. The primary composite endpoint was the occurrence of at least one of the combined major adverse clinical events (MACE) defined as cardiovascular mortality or nonfatal myocardial infarction (MI). The secondary endpoints were all-cause mortality, hospitalizations for heart failure (HF), late coronary revascularizations and sustained ventricular arrhythmias. All these clinical events were defined according to standardized definitions [14, 15], and are detailed in Additional file 2. Annualized event rates were expressed as the number of patients having the event as a proportion of the number of patients at risk divided by the number of patient-years follow-up. The adjudication of the cause of death between cardiovascular and non-cardiovascular was performed by two senior cardiologists (TP and MK), with a third cardiologist (JG) to reach final consensus. For patients who underwent percutaneous coronary intervention (PCI) < 90 days after index examination, the nine peri-procedural events (seven nonfatal MI or two CVD mortality) were excluded from the analysis.

### CMR protocol

The detailed stress CMR protocol has been previously published [16, 17], and is described in Additional file 3. Briefly, CMR was performed on a 1.5T CMR scanner (Siemens Healthineers, Erlangen, Germany). Vasodilation was induced with dipyridamole injected at 0.84 mg/kg during 3 min [18]. Then, a bolus of gadolinium-based contrast agent (Dotarem^®^, Guerbet LLC, France, 0.1 mmol/kg) was injected at a rate of 5.0 ml/s. Stress perfusion imaging was performed using an ECG-triggered saturation-prepared balanced steady-state free-precession sequence. A series of six slices (four short-axis views, a 2-chamber, and a 4-chamber view) were acquired every other heartbeat. No motion compensation was performed before analysis. Ten minutes after contrast injection, breath-hold contrast-enhanced 3D T1-weighted inversion-recovery gradient-echo sequence was acquired to detect late gadolinium enhancement (LGE). CMR sequence parameters are detailed in Additional file 4.

### CMR image analysis

Left ventricular (LV) end-diastolic volume (LVEDV), end-systolic volume (LVEDV) and systolic function were quantified on the short-axis cine stack. Stress perfusion and LGE images were evaluated according to the American Heart Association 17-segment model [19]. The analysis of perfusion images was done visually by two experienced physicians blinded to clinical and follow-up data. Inducible ischemia was defined as a subendocardial perfusion defect that (1) occurred in at least one myocardial segment, (2) persisted for at least three phases beyond peak contrast enhancement, (3) followed a coronary distribution, (4) in the absence of co-location with LGE in the same segment [11, 12]. An MI was defined by subendocardial or transmural LGE [20]. A myocardial segment was considered viable if LGE thickness was < 50% and nonviable when LGE thickness was ≥ 50% of the myocardial wall [21]. The total number of ischemic and LGE segments was assessed for each patient.

### CMR-related coronary revascularization

CMR-related coronary revascularization was defined as all procedures (coronary artery bypass grafting [CABG] or PCI performed within 90 days after stress CMR. All patients were treated with optimal medical therapy according to current guidelines in patients with chronic coronary syndromes [2]. Decision-making regarding initial coronary revascularization was based on the presence of myocardial ischemia in at least two contiguous segments in symptomatic patients, and the choice between PCI or CABG was made by the Heart Team of the Institutions. All clinical data, CMR parameters and CMR-related coronary revascularization were prospectively recorded into a dedicated database (Clinigrid software, Hemolia, France).

### Statistical analysis

Continuous variables were expressed as mean ± standard deviation (SD), categorical variables as frequency with a percentage, and follow-up as a median and interquartile range (IQR). Patients with and without inducible ischemia were compared using the Student’s t-test or the Wilcoxon rank-sum test for the continuous variables and the Chi-square or Fisher’s exact test for the categorical variables. Cumulative incidence rates of the outcomes were estimated using the Kaplan–Meier method and compared with the log-rank test. The data of patients who were lost to follow-up were censored to the time of the last contact. Cox proportional hazards methods were used to identify the predictors of MACE among patients with and without inducible ischemia. The assumption of the proportional hazards ratio (HR) was verified. To assess the incremental prognostic value of both the inducible ischemia and CMR-related coronary revascularization, different multivariable models were used, as follows:

- Model 1: used all clinical and CMR covariates for MACE and CV mortality with a p-value ≤0.1 on univariable screening (without ischemia and CMR-related coronary revascularization).
- Model 2a: model 1 with presence of inducible ischemia.
- Model 2b: model 1 with number of ischemic segments.
- Model 2c: model 1 with presence of ischemia with or without CMR-related coronary revascularization.

The discriminative capacity of each model for predicting MACE was determined according to Harrell’s C-statistic before and after the addition of inducible ischemia. The additional predictive value of the inducible ischemia was calculated using Harrell’s C-statistic increment. In addition, the global χ^2^ statistic was calculated for models with or without stress CMR parameters and compared using the likelihood ratio (LR) test for predicting MACE.

In the competitive risk analysis, cumulative incidence functions were used to display the proportion of patients with the event of interest or the competing event (nonfatal MI or CV mortality) as time progressed, and the Fine and Gray regression model was used for the sub-distribution hazard. A two-tailed p-value < 0.05 was considered statistically significant. Statistical analysis was performed using R software, version 3.3.1 (R Project for Statistical Computing, Vienna, Austria).

## Results

### Study population

**Fig. 1:**
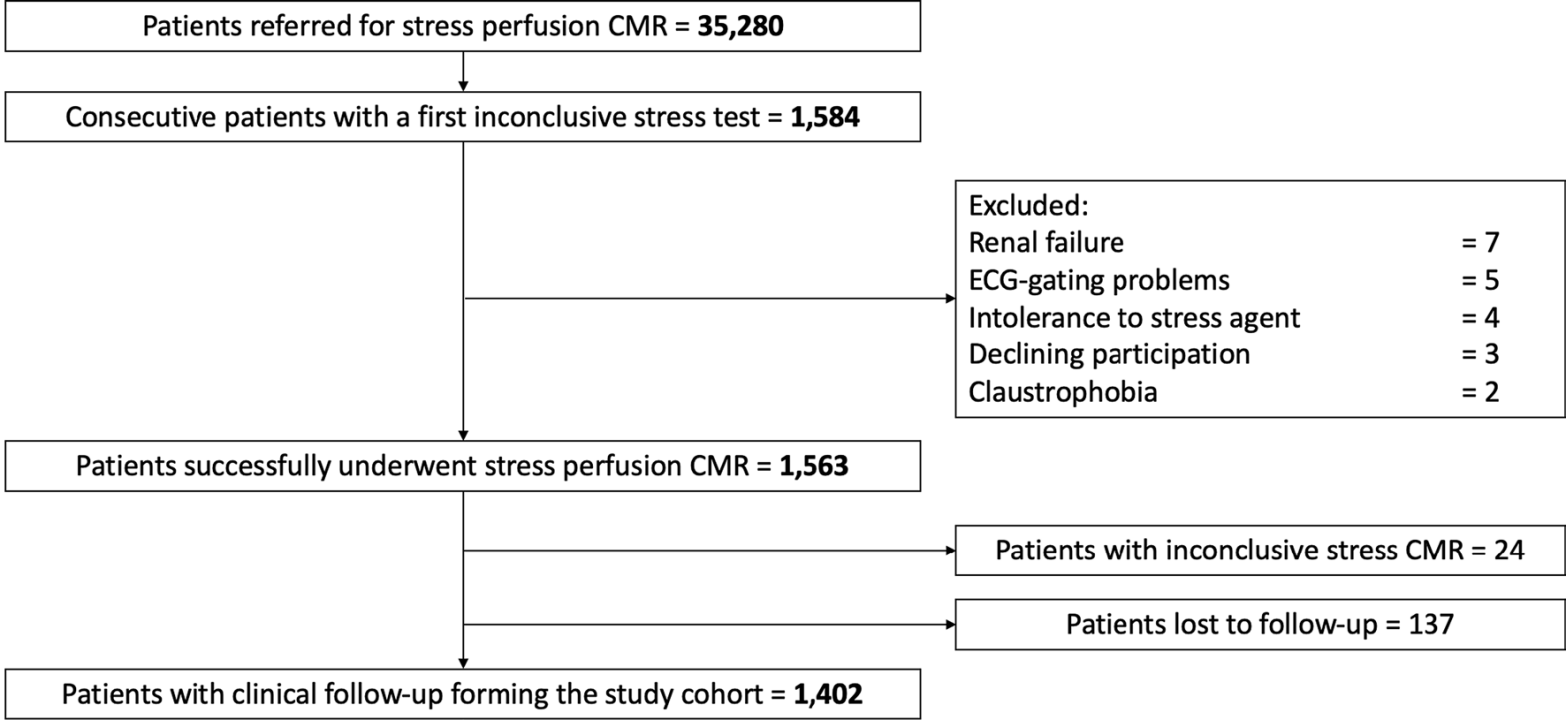
Flow diagram. *CMR* cardiovascular magnetic resonance; *ECG* electrocardigram

Of the 35,280 patients referred for stress CMR during the inclusion period, 1584 (4.5%) patients were referred for dipyridamole vasodilator stress CMR because of a first inconclusive noninvasive stress test. Among those, 1563 (98.7%) completed the stress protocol, as detailed in the flowchart (Fig. 1). Of the 1563 patients who successfully underwent stress CMR, the diagnosis of ischemia was inconclusive in 24 patients (1.5%) due to nondiagnostic image quality, arrythmias or artifacts. Out of these 1563 patients, 61 failed to respond to dipyridamole injection as assessed by the rate-pressure product (3.9%). No patient died during or shortly after CMR, and detailed safety results are presented in Additional file 5. Overall, 1402 patients completed the clinical follow-up and constituted our study cohort. Baseline subject characteristics and baseline CMR data are shown in Table 1. Among those 1402 patients (66.7% male, 69.5 ± 11.0 years), 58.4% had dyslipidemia, 57.6% had hypertension, 32.7% had diabetes mellitus, 30.7% had obesity, 27.9% had a family history of CAD and 24.0% were smokers. Overall, 727 (51.9%) patients had known CAD. Of note, 247 (17.6%) of patients were in atrial fibrillation or supraventricular arrhythmia. Regarding the first inconclusive stress test, 702 (50.1%) patients had a prior inconclusive stress echocardiography, 612 (43.7%) a prior inconclusive SPECT (147 dipyridamole SPECT) and 88 (6.3%) a prior inconclusive exercise ECG testing. The two main reasons for an inconclusive stress test were poor image quality (68%) and sub-maximal exercise (29%).

**Table 1:** Baseline and cardiovascular magnetic resonance (CMR) characteristics of patients with and without inducible ischemia on vasodilator stress CMR (N = 1402)

	All patients (N = 1402)	No inducible ischemia (N = 988)	Inducible ischemia (N = 414)	p value
Age, years	69.5 ± 11.0	68.5 ± 10.7	72.0 ± 11.3	**< 0.001**
Males, n (%)	935 (66.7)	641 (64.9)	294 (71.0)	**0.031**
Body mass index, kg/m^2^	28.6 ± 6.3	28.8 ± 6.5	28.4 ± 5.8	0.288
Body surface index, m^2^	2.0 ± 0.3	2.0 ± 0.3	2.0 ± 0.2	0.056
Coronary risk factors, n (%)				
Diabetes mellitus	459 (32.7)	297 (30.1)	162 (39.1)	**0.001**
Hypertension	807 (57.6)	542 (54.9)	265 (64.0)	**0.002**
Obesity^a^	431 (30.7)	313 (31.7)	118 (28.5)	0.266
Dyslipidemia	819 (58.4)	554 (56.1)	265 (64.0)	**0.007**
Smoking	336 (24.0)	248 (25.1)	88 (21.3)	0.142
Family history of CAD	391 (27.9)	268 (27.1)	123 (29.7)	0.358
Medical history of CVD, n (%)				
Known CAD	727 (51.9)	516 (52.2)	211 (51.0)	0.71
History of PCI	442 (31.5)	336 (34.0)	106 (25.6)	**0.002**
History of CABG	445 (31.7)	277 (28.0)	168 (40.6)	**< 0.001**
Known MI	340 (24.3)	246 (24.9)	94 (22.7)	0.42
Peripheral atheroma	158 (11.3)	72 (7.3)	86 (20.8)	**< 0.001**
Ischemic stroke	54 (3.9)	39 (3.9)	15 (3.6)	0.892
Pacemaker	7 (0.5)	4 (0.4)	3 (0.7)	0.428
Renal failure^b^	20 (1.4)	14 (1.4)	6 (1.4)	1.000
History of hospitalization for HF	52 (3.7)	42 (4.3)	10 (2.4)	0.133
Symptoms, n (%)				
Symptomatic angina	706 (50.4)	415 (42.0)	291 (70.3)	**< 0.001**
Dyspnea	211 (15.0)	158 (16.0)	53 (12.8)	0.149
High cardiovascular risk^c^	808 (57.6)	519 (52.5)	289 (69.8)	**< 0.001**
Ten-year risk for fatal CAD^d^, %	2.5 (1.2–5.9)	2.1 (0.8–5.3)	3.3 (1.5–6.4)	**< 0.001**
Indications to stress CMR, n (%)				
Inconclusive stress echocardiography	702 (50.1)	500 (50.6)	202 (48.8)	0.165
Inconclusive SPECT	612 (43.7)	438 (44.3)	174 (42.0)	0.159
Inconclusive exercise ECG testing	88 (6.3)	66 (6.7)	22 (5.3)	0.512
Cardiac rhythm, n (%)				
Sinus rhythm	1155 (82.4)	821 (83.1)	334 (80.7)	0.177
Atrial fibrillation/supraventricular arrhythmias	247 (17.6)	217 (22.0)	30 (7.2)	**< 0.001**
LV ejection fraction, %	50.4 ± 12.1	50.1 ± 11.9	51.2 ± 12.5	**0.007**
LV end-diastolic volume index, ml/m^2^	93.3 ± 31.2	94.8 ± 31.6	89.6 ± 30.1	**0.004**
LV end-systolic volume index, ml/m^2^	51.2 ± 26.1	52.5 ± 26.1	48.1 ± 26.0	**0.004**
LV mass, g/m^2^	73.9 ± 9.8	72.2 ± 9.8	78.7 ± 9.7	**< 0.001**
RV ejection fraction, %	63.6 ± 10.5	63.6 ± 10.5	63.5 ± 10.6	0.771
Presence of LGE, n (%)	556 (39.7)	422 (42.7)	134 (32.4)	**< 0.001**
Presence of viability if LGE, n (%)^e^	178 (12.7)	117 (11.8)	61 (14.7)	0.163
Number of LGE segments if LGE	0.9 ± 1.3	1.0 ± 1.3	0.8 ± 1.4	0.09
Number of ischemic segments	0.7 ± 1.2	0.0 ± 0.0	2.3 ± 1.2	**< 0.001**
RPP at baseline, mmHg/beats/min	9.1 (7.5–10.1)	9.1 (7.5–10.1)	9.2 (7.6–10.4)	0.533
RPP at stress, mmHg/beats/min	10.8 (10.4–12.7)	10.8 (10.4–12.7)	11.4 (9.9–14.5)	0.175
CMR-related coronary revascularization^b^, n (%)	323 (23.0)	0 (0)	323 (78.0)	**< 0.001**
By PCI	317 (22.6)	0 (0)	317 (76.6)	**< 0.001**
By CABG	6 (0.4)	0 (0)	6 (1.4)	**0.004**

Bold empasis means that the *P* value has reached statistical significance, with 2-tailed *P* value < 0.05

Values are n (%), mean ± SD, or median (interquartile range)

*BMI* body mass index, *CABG* coronary artery bypass graft, *CAD* coronary artery disease, *CMR* cardiovascular magnetic resonance, *CVD* cardiovascular disease, *ECG* electrocardiogram, *HF* heart failure, *LGE* late gadolinium enhancement, *LV* left ventricle, *MI* myocardial infarction, *PCI* percutaneous coronary intervention, *RPP* rate-pressure product (pressure mmHg × Heart rate bpm)/1000, *SD* standard deviation

^a^Defined by BMI ≥ 30 kg/m^2^

^b^Defined by glomerular filtration rate < 60 ml/min/1.73 m^2^

^c^Defined by Framingham Risk Score > 20% of risk of CAD at 10 years

^d^Based on a modified SCORE project (https://www.escardio.org/Education/Practice-Tools/CVD-prevention-toolbox/SCORE-Risk-Charts) that did not take into account the total cholesterol level [22, 23]

^e^Defined by the presence of LGE with < 50% transmurality

Among the 1402 patients, 485 (34.6%) were asymptomatic without angina or dyspnea. These asymptomatic patients were older and had a higher rate of known CAD (87.0% vs 33.3%, p < 0.001), diabetes mellitus, hypertension and smoking than symptomatic patients with angina or dyspnea (Additional file 6). Consistently, asymptomatic patients presented a higher CV risk than symptomatic using the ESC SCORE 10-year risk for fatal CAD [22] (3.4 [2.2–6.9] vs 2.1 [0.7–5.5] %, p < 0.001).

The study cohort had a mean LV ejection fraction (LVEF) of 50.4 ± 12.1%. LGE was present in 556 (39.7%) and presence of inducible ischemia was detected in 414 (29.5%) patients with a mean extent of 2.3 ± 1.2 segments (Additional file 7). Of note, the rate of inducible ischemia in the overall population of 35,280 patients referred for stress CMR during the inclusion period was 12.4%.

Patients with inducible ischemia were older, more frequently males and had a higher rate of diabetes mellitus, hypertension, dyslipidemia and history of peripheral atheroma than patients without inducible ischemia (Table 1). Of 414 patients with ischemia, 381 (92.0%) had a coronary angiography. Among those, 323 (84.8%) underwent CMR-related coronary revascularization (317 [98.1%] PCI and 6 [1.9%] CABG).

### Cardiovascular events

**Fig. 2:**
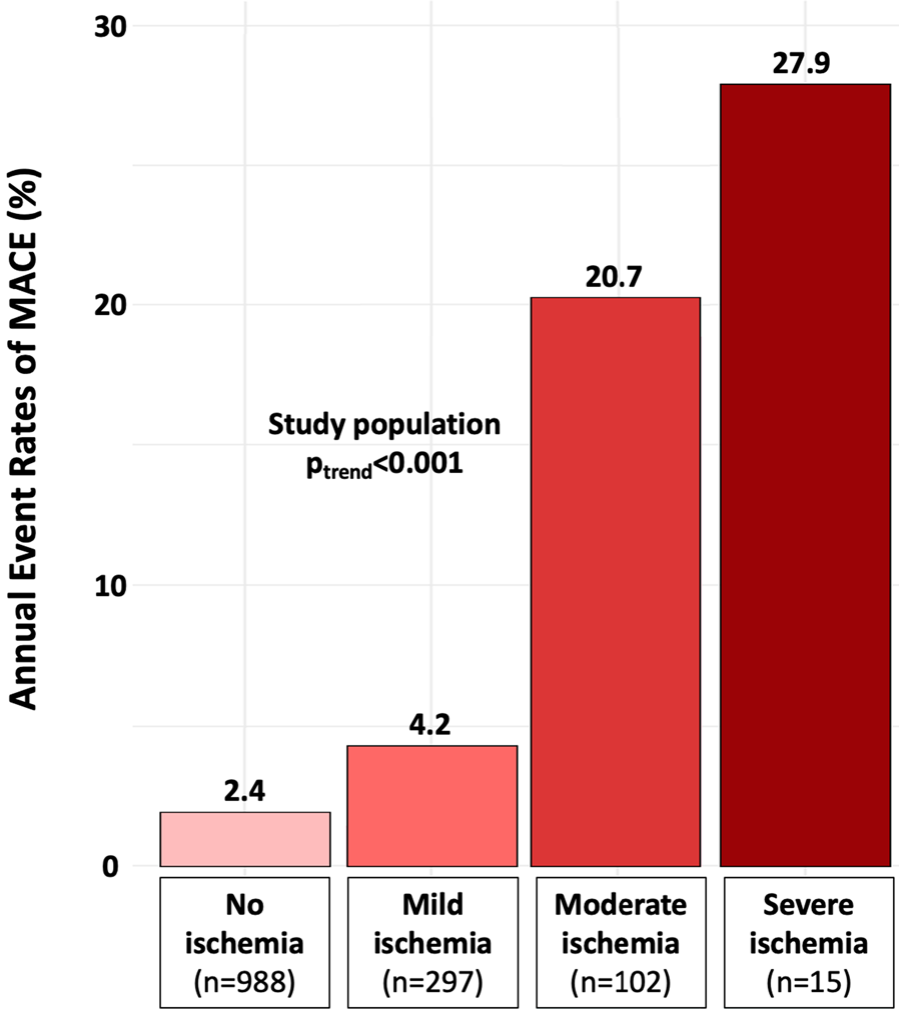
Annualized rates of major adverse cardiovascular events (MACE) stratified by the extent of myocardial ischemia. Annualized rates of MACE stratified by the extent of myocardial ischemia. Mild, moderate, and severe ischemia were defined as the involvement of 1–2, 3–5, and ≥ 6 myocardial segments, respectively. Test comparing the groups was based on the Cochran–Armitage test for trend

During a median follow-up of 6.5 (IQR 5.6–7.5) years, there were 197 (14.1%) MACE, including 141 (10.1%) CV mortality and 73 (5.2%) nonfatal MI. Furthermore, 255 all-cause mortality (18.2%), 106 HF hospitalisations (7.6%), 99 late coronary revascularizations (7.1%), and 34 sustained documented ventricular arrhythmias (2.4%). Annualized event rates were 4.4% for MACE, 2.4% for CVD mortality, and 4.6% for all-cause mortality. Patients without inducible ischemia or LGE had the lowest annualized rate of MACE (2.1%/year), whereas the annualized rate of MACE was greater for patients with inducible ischemia without or with LGE (9.0%/year and 9.3%/year respectively, both p < 0.001) (Additional file 8). The annualized rate of MACE was lower in patients without inducible ischemia compared to patients with mild, moderate, or severe ischemia (2.4%/year vs. 4.2%/year, 20.7%/year and 27.9%/year, respectively; p_trend_ < 0.001) (Fig. 2). In addition, the prognostic value of the presence of inducible ischemia was consistent irrespective of age (Additional file 9).

### Prognostic value of stress CMR parameters

**Fig. 3:**
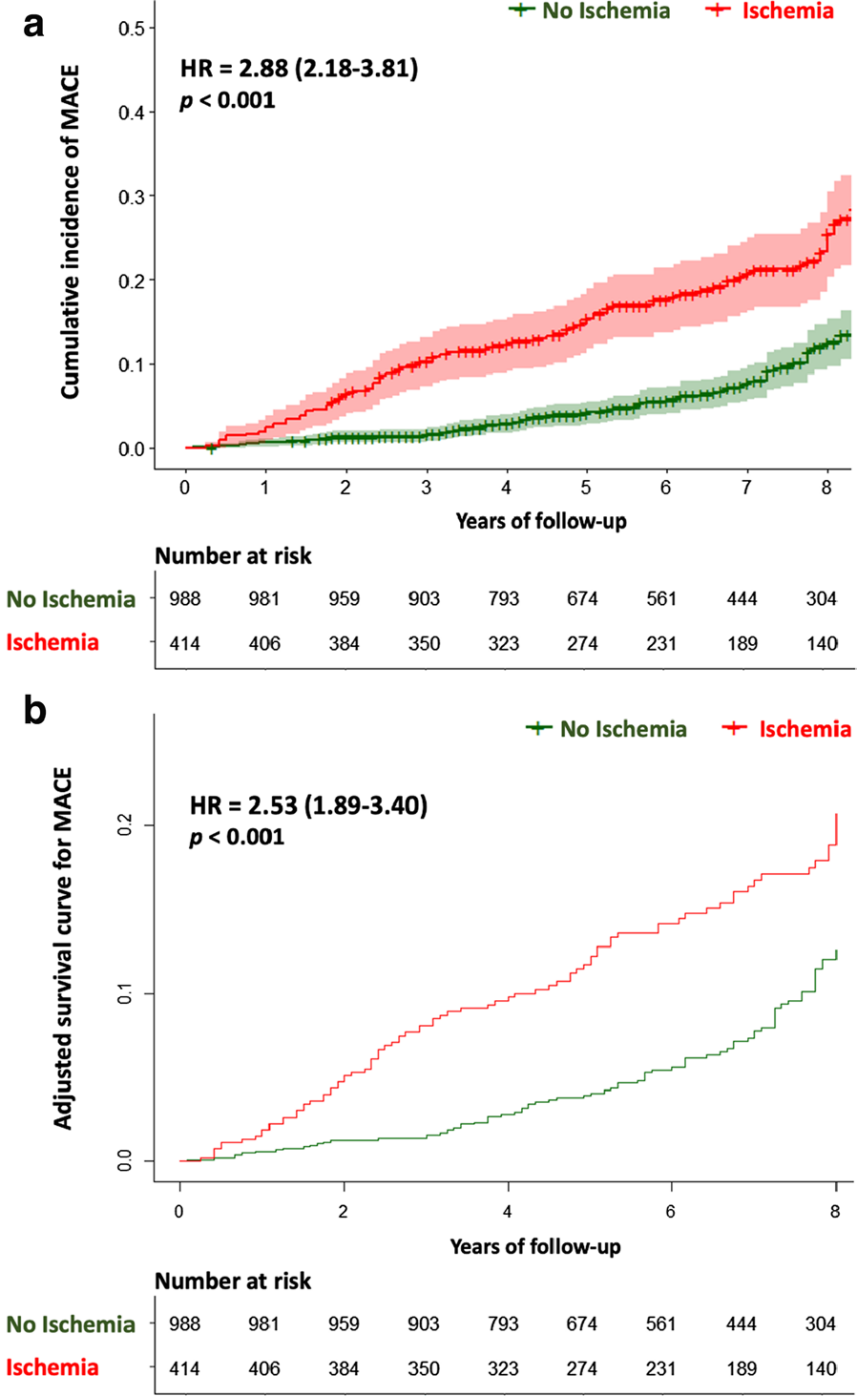
Survival curves for MACE stratified by the presence of inducible ischemia. The univariable analysis for MACE (**a**) was performed using the log-rank test to compare patients with ischemia and without ischemia. The adjusted survival curve for MACE (**b**) was performed with the final model including: age, male, hypertension, diabetes, dyslipidemia, known CAD, LVEF per 10%, LV end-diastolic volume index, modified SCORE project, the presence of LGE and the presence of ischemia. HR indicates hazard ratio

In univariable analysis, age, male gender, hypertension, diabetes mellitus, dyslipidemia, known CAD, LVEF value, LV end-diastolic volume (LVEDV) and end-systolic volume (LVESV) indexed (LVEDVI, LVESVI, respectively) and the presence and extent of both inducible ischemia and LGE were all significantly associated with MACE (Table 2). Using Kaplan–Meier analysis, the presence of inducible ischemia was associated with the occurrence of MACE (HR: 2.88, 95% CI 2.18–3.81, Fig. 3) and CV mortality (HR: 2.44 95% CI 1.75–3.40; both p < 0.001), and the same finding was observed for LGE (HR: 1.46, 95% CI 1.16–1.89; and HR: 1.38, 95% CI 1.06–1.77, both p < 0.001; respectively). In the overall population, the CMR-related coronary revascularization was associated with the occurrence of MACE and CV mortality (HR: 2.43 95% CI 1.83–3.22; and HR: 2.04 95% CI 1.46–2.86; respectively both p < 0.001). The prognostic value of inducible ischemia to predict MACE was consistent for both women and men (Additional file 10); and both asymptomatic and symptomatic patients (Additional file 11).

**Table 2:** Univariable analysis of clinical and CMR characteristics for prediction of adverse events

	MACE		Cardiovascular mortality	
	Hazard ratio (95% CI)	p value	Hazard ratio (95% CI)	p value
Age	1.04 (1.02–1.05)	**< 0.001**	1.06 (1.04–1.08)	**< 0.001**
Male	1.56 (1.14–2.14)	**0.006**	1.77 (1.21–2.60)	**0.003**
BMI	1.04 (0.98–1.09)	0.123	0.96 (0.90–1.03)	0.282
Hypertension	1.22 (1.03–1.60)	**0.033**	1.18 (0.97–1.57)	0.077
Diabetes mellitus	1.35 (1.04–1.77)	**0.041**	1.59 (1.14–2.23)	**0.006**
Dyslipidemia	1.41 (1.05–1.89)	**0.022**	1.55 (1.09–2.20)	**0.014**
Smoking	1.02 (0.77–1.36)	0.757	1.03 (0.79–1.40)	0.675
Family history of CAD	1.01 (0.74–1.38)	0.937	1.05 (0.73–1.50)	0.810
Known CAD	1.30 (1.01–1.69)	**0.043**	1.21 (0.93–1.71)	0.122
Known MI	0.80 (0.57–1.15)	0.222	0.82 (0.53–1.26)	0.361
Peripheral atheroma	1.35 (0.84–2.17)	0.212	1.59 (0.94–2.67)	0.082
Ischemic stroke	1.03 (0.53–2.01)	0.936	0.90 (0.39–2.03)	0.793
History of hospitalization for HF	1.35 (0.69–2.64)	0.376	1.48 (0.69–3.17)	0.311
Symptomatic angina	1.63 (0.83–3.22)	0.16	0.92 (0.50–1.71)	0.88
Dyspnea	0.91 (0.47–1.73)	0.77	0.78 (0.47–1.30)	0.35
Ten-year risk for fatal CAD (modified SCORE project)	1.22 (1.08–1.80)	**< 0.001**	1.29 (1.10–1.83)	**< 0.001**
Presence of inducible ischemia	2.88 (2.18–3.81)	**< 0.001**	2.44 (1.75–3.40)	**< 0.001**
Number of segments of inducible ischemia	1.61 (1.51–1.72)	**< 0.001**	1.55 (1.43–1.67)	**< 0.001**
Presence of LGE	1.46 (1.16–1.89)	**< 0.001**	1.38 (1.06–1.77)	**0.031**
Number of segments of LGE	1.40 (1.30–1.52)	**< 0.001**	1.33 (1.21–1.45)	**< 0.001**
LVEF^a^	0.85 (0.79–0.91)	**< 0.001**	0.92 (0.84–0.98)	**0.024**
LV end-diastolic volume index^a^	1.07 (1.04–1.10)	**< 0.001**	1.03 (0.90–1.37)	0.315
LV end-systolic volume index^a^	1.09 (1.05–1.12)	**< 0.001**	1.05 (0.92–1.42)	0.221
RV ejection fraction	0.99 (0.80–1.23)	0.45	1.09 (0.81–1.52)	0.72
CMR-related coronary revascularization	2.43 (1.83–3.22)	**< 0.001**	2.04 (1.46–2.86)	**< 0.001**

Bold empasis means that the *P* value has reached statistical significance, with 2-tailed *P* value < 0.05

*BMI* body mass index, *CAD* coronary artery disease, *CI* confidence interval, *CMR* cardiac magnetic resonance, *HF* heart failure, *LGE* late gadolinium enhancement, *LV* left ventricle, *LVEF* left ventricular ejection fraction, *MACE* major adverse cardiac events, *MI* myocardial infarction, *RV* right ventricle

^a^Increment of 10 units

^b^Defined by a coronary revascularisation performed within 90 days after the stress CMR examination

**Fig. 4:**
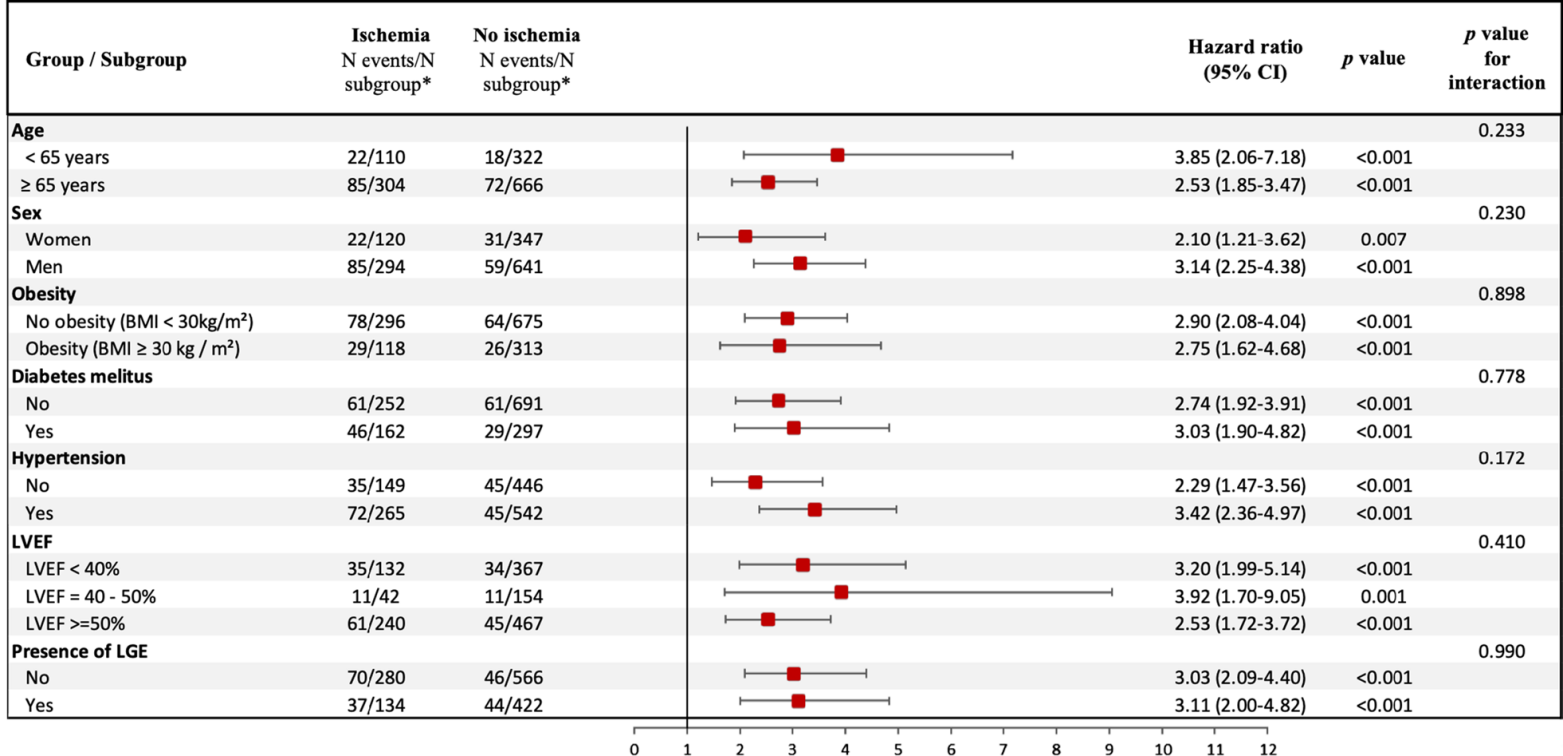
Subgroup analysis. Forest-plot of incidence of MACE based on the presence of myocardial ischemia in specified subgroups. *N events/N subgroup: number of patients had a major adverse clinical events (MACE)/number of patients in the subgroup

In the overall population, inducible ischemia was also associated with nonfatal MI (HR: 5.04, 95% CI 3.09–8.21; p < 0.001), late elective coronary revascularization (HR: 2.61, 95% CI 1.76–3.87; p < 0.001), ventricular arrythmias (HR: 2.76, 95% CI 1.41–5.42; p = 0.003), and all-cause mortality (HR: 1.73, 95% CI 1.35–2.22; p < 0.001) (Additional file 12. The prognostic value of inducible ischemia remained consistent in different subgroups of clinical interest, such as diabetics and non-diabetics, obese and non-obese, and regardless of LVEF value or the presence of LGE (Fig. 4).

**Fig. 5:**
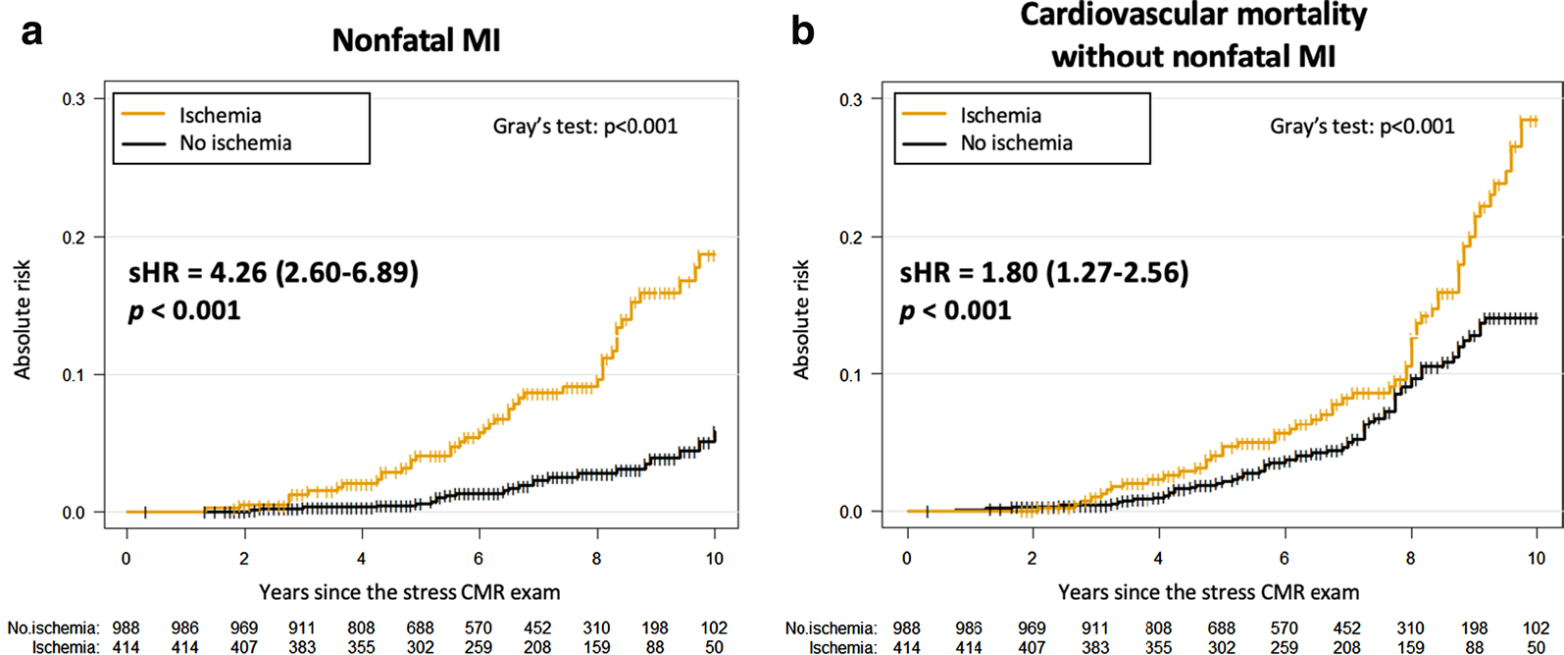
Competing risk analysis for nonfatal MI and cardiovascular mortality stratified by presence of inducible ischemia. Cumulative incidence functions of nonfatal MI (**a**) or cardiovascular mortality without nonfatal MI (**b**). Test comparing the groups was based on the Fine and Gray’s test for trend

In multivariable stepwise Cox regression analysis, age, male gender, the presence of inducible ischemia and the number of ischemic segments were independent predictors of a higher incidence of MACE (HR: 1.05, 95% CI 1.03–1.06, p < 0.001; HR: 1.47, 95% CI 1.05–2.06, p = 0.027; HR: 2.53, 95% CI 1.89–3.40, p < 0.001; and HR: 1.58, 95% CI 1.47–1.71, p < 0.001; respectively) (Table 3). In competitive risk analysis, the presence of inducible ischemia was independently associated with nonfatal MI and CV mortality (HR: 4.26, 95% CI 2.60–6.89, p < 0.001 and HR: 1.80, 95% CI 1.27–2.56, p < 0.001; respectively) (Fig. 5 and Additional file 13).

**Table 3:** Multivariable Cox regression analysis for the prediction of adverse events

	MACE		Cardiovascular mortality	
	Hazard ratio (95% CI)	p value	Hazard ratio (95% CI)	p value
Model 1^a^				
Age	1.05 (1.03–1.06)	**< 0.001**	1.07 (1.05–1.10)	**< 0.001**
Male	1.49 (1.07–2.07)	**0.002**	1.78 (1.17–2.68)	**< 0.001**
Hypertension	1.10 (0.97–1.28)	0.098	–	–
Diabetes	1.34 (0.97–1.81)	0.061	1.50 (1.05–2.16)	**0.030**
Dyslipidemia	1.32 (0.98–1.83)	0.050	1.40 (0.97–1.99)	0.073
Known CAD	1.17 (0.84–1.65)	0.354	1.27 (0.85–1.89)	0.238
LVEF	0.91 (0.80–1.03)	0.094	0.90 (0.78–1.02)	0.098
Presence of LGE	1.07 (0.77–1.48)	0.691	0.90 (0.80–1.02)	0.104
LV end-diastolic volume index	1.02 (0.92–1.20)	0.082	–	–
Ten-year risk for fatal CAD (modified SCORE project)	1.05 (0.84–2.17)	0.332	1.19 (0.90–2.43)	0.182
Model 2a^b^				
+ Presence of inducible ischemia	2.53 (1.89–3.40)	**< 0.001**	1.83 (1.29–2.60)	**< 0.001**
Model 2b^c^				
+ Number of ischemic segments	1.58 (1.47–1.71)	**< 0.001**	1.44 (1.32–1.57)	**< 0.001**
Model 2c^d^				
+ Presence of ischemia without revascularization	2.88 (1.82–4.56)	**< 0.001**	2.23 (1.30–3.85)	**0.004**
+ Presence of ischemia with revascularization	2.44 (1.79–3.34)	**< 0.001**	1.74 (1.20–2.52)	**0.003**

Bold empasis means that the *P* value has reached statistical significance, with 2-tailed *P* value < 0.05

*CAD* coronary artery disease, *CI* confidence interval, *CMR* cardiovascular magnetic resonance, *LGE* late gadolinium enhancement, *MACE* major adverse cardiac events, *LVEF* left ventricular ejection fraction

^a^Covariates in the model 1 by stepwise variable selection with entry and exit criteria set at the p ≤ 0.1 level: (1) For MACE: age, male, hypertension, diabetes, dyslipidemia, known CAD, LVEF per 10%, LV end-diastolic volume index, modified SCORE project and the presence of LGE. (2) For CV mortality: age, male, diabetes, dyslipidemia, known CAD, LVEF per 10%, modified SCORE project and the presence of LGE

^b^Covariates in the model 2a: model 1 with presence of ischemia

^c^Covariates in the model 2b: model 1 with number of ischemic segments

^d^Covariates in the model 2c: model 1 with presence of ischemia with or without CMR-related coronary revascularization. This variable was defined in three categories: presence of ischemia without CMR-related coronary revascularization, presence of ischemia with CMR-related coronary revascularization and absence of ischemia (reference for hazard ratio calculations)

**Fig. 6:**
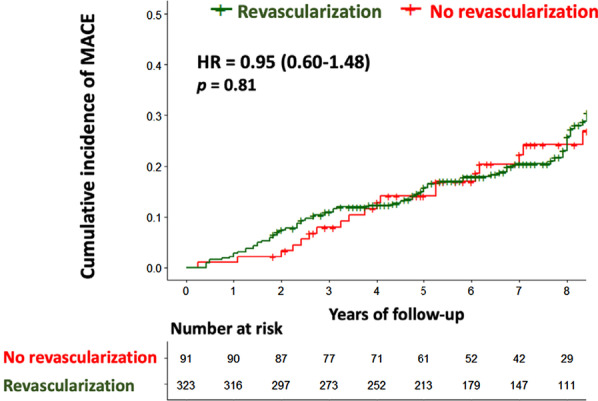
Kaplan–Meier curves for MACE stratified by presence/absence of stress CMR-related coronary revascularization in patients with inducible ischemia. Kaplan Meier curves for MACE as a function of length of follow-up for patients with or without early CMR-related coronary revascularization within 90 days after CMR in patients with inducible ischemia. Test comparing the two groups was based on the log-rank test

Using Kaplan–Meier analysis only in patients with inducible ischemia, CMR-related coronary revascularization was not associated with the occurrence of MACE (HR: 0.95, 95% CI 0.60–1.48; p = 0.81) (Fig. 6). Inducible ischemia remained independently associated with MACE in patients without or with coronary revascularization (HR: 2.88, 95% CI 1.82–4.56; and HR: 2.44, 95% CI 1.79–3.34, both p < 0.001; respectively) (Table 3).

### Incremental prognostic value of stress CMR

For the prediction of MACE, the baseline C-statistic value was 0.63 (95% CI 0.60–0.68) for model 1 with stepwise variable selection. The addition of inducible ischemia or the number of ischemic segments significantly improved the C-statistic to 0.73 (95% CI 0.66–0.79; C statistic improvement for model 1: 0.10) and 0.75 (95% CI 0.69–0.81; C statistic improvement for model 1: 0.12), respectively. Furthermore, the addition of both presence of inducible ischemia and CMR-related coronary revascularization did not improve the C-statistic compared to the model with only the presence of inducible ischemia (C-statistic 0.73 for both) (Table 4).

**Table 4:** Discrimination associated with the CMR-parameters for the prediction of MACE

	MACE		
	C-index (95%CI)	Global χ^2^ statistic	LR-test
Model 1^a^ (stepwise selection)	0.63 (0.60–0.68)	420.1	Reference
Model 2a^b^ (model 1 + presence of ischemia)	0.73 (0.66–0.79)	580.9	< 0.001
Model 2b^c^ (model 1 + number of ischemic segments)	0.75 (0.69–0.81)	607.4	< 0.001
Model 2c^d^ (model 1 + ischemia with or without CMR-related revascularization)	0.73 (0.66–0.80)	570.2	< 0.001

*BMI* body mass index, *CAD* coronary artery disease, *CI* confidence interval, *CV* cardiovascular, *HF* heart failure, *LGE* late gadolinium enhancement, *LVEF* left ventricular ejection fraction, *MACE* major adverse cardiac events

^a^Covariates in the model 1 by stepwise variable selection with entry and exit criteria set at the p ≤ 0.1 level: age, male, hypertension, diabetes, dyslipidemia, known CAD, LVEF per 10% and presence of LGE

^b^Covariates in the model 2a: model 1 with presence of ischemia

^c^Covariates in the model 2b: model 1 with number of ischemic segments

^d^Covariates in the model 2c: model 1 with presence of ischemia with or without CMR-related coronary revascularization. This variable was defined in three categories: presence of ischemia without CMR-related coronary revascularization, presence of ischemia with CMR-related coronary revascularization and absence of ischemia (reference for hazard ratio calculations)

## Discussion

In this study of consecutive series of patients with a first inconclusive noninvasive stress test referred for vasodilator stress CMR, the main findings are: (1) 29.5% of patients had inducible ischemia and 14.1% had MACE after median follow-up of 6.5 years; (2) inducible ischemia and LGE were long-term predictors of MACE and CVD mortality; (3) the presence and extent of inducible ischemia were independently associated with MACE and CV mortality; (4) the presence or extent of inducible ischemia improved model discrimination for the prediction of MACE, after adjusting for traditional CV risk factors; (5) there was no benefit of CMR-related coronary revascularization in reducing MACE.

The prevalence of inducible ischemia (29.5%) and LGE (39.7%) are consistent with previous large studies in patients with suspected or known CAD [11, 12, 22]. The rate of MACE reported over the follow-up period (14.1%) is in line with contemporary cohorts of patients referred for stress CMR [11, 23], a meta-analysis of patients with inconclusive stress echocardiography [24], and the ISCHEMIA trial [25]. Notably, the rate of inducible ischemia in patients referred for inconclusive stress test was higher (29.5%) than in the overall population of 35,280 patients referred for stress CMR during the same inclusion period (12.4%). Besides, the global annualized events rate (4.4%/year) of this study was higher than the annualized rate described in patients with normal CMR in previous larges studies (1%/year) [12, 23]. This finding is consistent with a recent study showing that patients with inconclusive stress tests had a higher rate of CV events compared with those with negative results [6].

Although the long-term prognostic value of stress CMR is well established in patients with known or suspected CAD [11, 12, 26], there is no prognostic data in patients with a first inconclusive stress test [7]. In the current study, the presence of inducible ischemia and LGE were associated with MACE and CVD mortality. In accordance with some recent studies [22, 27], the extent of inducible ischemia was a strong and independent predictor of MACE and CVD mortality. In agreement with previous functional imaging studies [28, 29], the extent of inducible ischemia assessed by stress CMR had the best incremental prognostic value in predicting MACE, with better discrimination over traditional risk factors than the sole presence of inducible ischemia.

We found the prognostic value of stress CMR for predicting MACE was significant for both symptomatic and asymptomatic patients. Interestingly, asymptomatic patients addressed after a first inconclusive stress test had known CAD in the vast majority of cases (87%) or at high CVD risk (13%). Because patients with silent myocardial ischemia have at least similar risk for CVD events and mortality than symptomatic patients with typical angina [30, 31] risk stratification of asymptomatic patients may be useful in managing secondary prevention. Although the current guidelines do not recommend systematic stress testing in the work-up of patients with CAD [2, 3], the current data demonstrate a significant prognostic value of stress CMR in asymptomatic patients.

The rate of CMR-related revascularization was 78.0% in patients with inducible ischemia, which is consistent with recent studies [12, 27, 29]. In line with the ISCHEMIA trial that recently showed the lack of benefit of coronary revascularization in reducing MACE in patients with stable coronary disease [25], the current study suggests no association between CMR-related coronary revascularization and improved outcome.

While the current guidelines recommend to perform an additional noninvasive testing (class IIa) in patients with a first inconclusive stress test [2, 3], this strategy is used in < 25% of the cases resulting in significant economic implications with increased healthcare costs [4, 6]. Interestingly, a report from the SPINS registry of the Society for Cardiovascular Magnetic Resonance [12] has recently demonstrated that the average cost of ischemic testing was lower for stress CMR than nuclear stress or the use of initial coronary angiography [32]. The current study demonstrates that an improved risk stratification using stress CMR could allow to identify high-risk patients who could benefit from treatment intensification and new therapies. Future studies should prospectively randomize some diagnostic algorithms following an inconclusive stress test to define optimal testing strategies.

## Study limitations

First, the study was retrospective with 8.8% patients lost to follow-up, which can be explained by the relatively long follow-up period. The analysis of the CMR perfusion scans was visual, but it represents the most widely accepted clinical method with optimal diagnostic accuracy. Because of the lack of randomization, the prognostic impact of CMR-related revascularization cannot be formally established. Moreover, the reasons for the absence of revascularization in patients with ischemia mostly included non-significant lesions, technical difficulties, small ischemic territory, and coronary arteries < 2 mm diameter, but those data were not formally collected. Also, technical details regarding revascularization such as the type and number of stents or anti-platelet strategy were not collected. However, these limitations were related to patient care and reflect current clinical practice. The Syntax score or other specific predictive models of CVD events after revascularization were not available in this study. The current study was not designed to assess the potential prognostic value of a first inconclusive stress test before stress CMR. Finally, although adenosine or regadenoson is commonly used for vasodilator stress CMR, dipyridamole was used in our center mainly because of medico-economic reasons and very close efficacy/safety profile compared to adenosine.

## Conclusion

In consecutive patients with a first inconclusive noninvasive stress test, stress perfusion CMR has good long-term prognostic value to predict MACE and CV mortality. The presence and extent of inducible myocardial ischemia are independently associated with CV mortality and nonfatal MI and offer incremental prognostic value over traditional CVD risk factors.

## Supplementary Information

**Additional file 1.** Definitions of positive and negative stress tests.

**Additional file 2.** Definition of clinical events.

**Additional file 3.** CMR protocol and analysis.

**Additional file 4.** Table. CMR sequence parameters.

**Additional file 5.** Safety results.

**Additional file 6.** Table. Baseline characteristics of patients according to the presence of symptoms.

**Additional file 7.** Figure. Examples of inducible myocardial ischemia on stress CMR in patients with a prior inconclusive stress test.

**Additional file 8.** Figure. Annualized rates of MACE stratified by the presence of myocardial ischemia and late gadolinium enhancement (LGE).

**Additional file 9.** Figure. Age interaction: annualized event rates of MACE stratified by age and presence/absence of myocardial ischemia.

**Additional file 10.** Figure. Kaplan-Meier Curves for MACE stratified by the presence of ischemia and according to patient sex.

**Additional file 11.** Figure. Kaplan-Meier Curves for MACE stratified by the presence of ischemia and according to the presence of symptoms.

**Additional file 12.** Table. Univariable and multivariable analysis of CMR-induced myocardial ischemia for prediction of adverse events.

**Additional file 13.** Table. Univariable and Multivariable Competing Risk Regression Analysis.

## Data Availability

All data generated or analysed during this study are included in this published article [and its additional information files].

## Abbreviations

BMI: Body mass index
CAD: Coronary artery disease
CABG: Coronary artery bypass grafting
CMR: Cardiovascular magnetic resonance
CTA: Computed tomography angiography
CVD: Cardiovascular disease
ECG: Electrocardiogram
HF: Heart failure
LGE: Late gadolinium enhancement
LV: Left ventricle/left ventricular
LVEDV: Left ventricular end-diastolic volume
LVEDVI: Left ventricular end-distolic volume index
LVEF: Left ventricular ejection fraction
LVESV: Left ventricular end-systolic volume
LVESVI: Left ventricular end-systolic volume index
MACE: Major adverse clinical events
MI: Myocardial infarction
PCI: Percutaneous coronary intervention
RPP: Rate pressure product
SPECT: Single photon emission computed tomography
