# Supplementary material for: Prognostic value of vasodilator stress perfusion cardiovascular magnetic resonance after inconclusive stress testing
Source: J Cardiovasc Magn Reson. 2021 Jul 5;23:89. doi: 10.1186/s12968-021-00785-6 (PMC8256486; doi:10.1186/s12968-021-00785-6)
Supplement: Supplementary file 1 — Additional file 1. Definitions of positive and negative stress tests. [file 12968_2021_785_MOESM1_ESM.docx]

**ADDITIONAL FILE 1**

**Definitions of positive and negative stress tests**

1. ***Definition of positive stress test***

A positive exercise electrocardiogram (ECG) (non-imaging) was defined as ST-segment changes consistent with ischemia during stress or early termination (< 3 minutes) due to symptom reproduction, hypotension, or arrhythmia.

Positive stress nuclear and stress echocardiography testing were defined as inducible ischemia in at least one of either anterior, inferior, or lateral territory corresponding to an expected, left anterior descending, right coronary, or left circumflex artery distribution or if an exercise stress test was terminated early (< 3 minutes) due to ST-segment changes consistent with ischemia, symptom reproduction, hypotension, or arrhythmia.

1. ***Definition of negative stress test***

A negative test was defined as the absence of the above criteria in an otherwise technically conclusive study. In addition, exercise ECG testing was considered to be inconclusive if the stress ECG was deemed borderline or indeterminate due to poor technical quality, left bundle branch block, or submaximal heart rate (<85% maximal predicted heart rate).
