# Supplementary material for: Prognostic value of vasodilator stress perfusion cardiovascular magnetic resonance after inconclusive stress testing
Source: J Cardiovasc Magn Reson. 2021 Jul 5;23:89. doi: 10.1186/s12968-021-00785-6 (PMC8256486; doi:10.1186/s12968-021-00785-6)
Supplement: Supplementary file 2 — Additional file 2. Definition of clinical events. [file 12968_2021_785_MOESM2_ESM.docx]

**ADDITIONAL FILE 2**

**Definition of all clinical events**

Nonfatal myocardial infarction (MI) was defined by typical angina of ≥ 20 min duration, ECG changes, and a rise in troponin or creatine kinase level above the 99 percentile of the upper reference limit^1^. Cardiovascular mortality was defined as sudden cardiac death with documented fatal arrhythmias or any death immediately preceded by acute MI, acute or exacerbation of heart failure, or stroke. All-cause mortality was defined using the electronic French National Registry of Death (*Institut National de la Statistique et des Etudes Economiques*, INSEE registry). All clinical events were defined according to standardized definitions^2^. Late coronary revascularization was defined by a revascularization occurring > 90 days after CMR. For patients who underwent PCI within 90 days after the index examination, peri-procedural events (MI or cardiovascular mortality)^3^ were not included in the analysis.

**References**

1. Thygesen K, Alpert JS, Jaffe AS, Chaitman BR, Bax JJ, Morrow DA, White HD, Executive Group on behalf of the Joint European Society of Cardiology (ESC)/American College of Cardiology (ACC)/American Heart Association (AHA)/World Heart Federation (WHF) Task Force for the Universal Definition of Myocardial Infarction. Fourth Universal Definition of Myocardial Infarction (2018). *Circulation*. 2018;138:e618–e651.

2. Hicks KA, Tcheng JE, Bozkurt B, Chaitman BR, Cutlip DE, Farb A, Fonarow GC, Jacobs JP, Jaff MR, Lichtman JH, Limacher MC, Mahaffey KW, Mehran R, Nissen SE, Smith EE, Targum SL. 2014 ACC/AHA Key Data Elements and Definitions for Cardiovascular Endpoint Events in Clinical Trials. *Journal of the American College of Cardiology*. 2015;66:403–469.

3. Stone GW, Ben-Yehuda O, Sabik JF, Kappetein AP, Serruys PW. Considerations for an optimal definition of procedural myocardial infarction. *Eur Heart J*. 2020;41:1704–1705.
