# Supplementary material for: Prognostic value of vasodilator stress perfusion cardiovascular magnetic resonance after inconclusive stress testing
Source: J Cardiovasc Magn Reson. 2021 Jul 5;23:89. doi: 10.1186/s12968-021-00785-6 (PMC8256486; doi:10.1186/s12968-021-00785-6)
Supplement: Supplementary file 3 — Additional file 3. CMR protocol and analysis. [file 12968_2021_785_MOESM3_ESM.docx]

**ADDITIONAL FILE 3**

**Cardiovascular magnetic resonance (CMR) Protocol**

CMR was performed in a dedicated CMR laboratory on a 1.5T scanner (MAGNETOM Espress, Siemens Healthineers, Erlangen, Germany) from December 2008 to August 2015 and MAGNETOM Aera (Siemens Healthineers) from August 2015 to January 2020) with an 18-channel anterior phased-array coil. Long-axis (2-, 3-, and 4-chamber) and short-axis cine images encompassing the left ventricle from base to apex were obtained with a segmented retrospectively gated balanced steady-state free-precession (bSSFP) sequence. Vasodilation was induced with dipyridamole injected at 0.84 mg/kg over 3 min. Then, a bolus of gadolinium-based contrast agent (Dotarem^®^, Guerbet, France, 0.1 mmol/kg) was injected at a rate of 5.0 ml/s with an injector (Mallinckrodt Optistar^®^ Elite). Stress perfusion imaging was performed using an ECG-triggered saturation-prepared bSSFP sequence. A series of six slices (four short-axis views, a 2-chamber, and a 4-chamber view) were acquired every other heartbeat. Then, theophylline was injected intravenously to null the effect of dipyridamole. Ten minutes after contrast injection, breath-hold contrast-enhanced 3D T1-weighted inversion-recovery gradient-echo sequences were acquired to detect late gadolinium enhancement (LGE). The inversion time was individually adjusted to null normal myocardium. Patients were asked to refrain from caffeine at least 12 h before CMR. Safety was studied with clinical monitoring 1 h after CMR. A 12-lead ECG was performed before and after CMR examination.

The *syngo*.via software (Siemens Healthineers) was used for image display and processing, and Hemolia (Clinigrid Inc., Paris, France) was used for reporting.
