# Supplementary material for: Prognostic value of vasodilator stress perfusion cardiovascular magnetic resonance after inconclusive stress testing
Source: J Cardiovasc Magn Reson. 2021 Jul 5;23:89. doi: 10.1186/s12968-021-00785-6 (PMC8256486; doi:10.1186/s12968-021-00785-6)
Supplement: Supplementary file 4 — Additional file 4. Table. CMR sequence parameters. [file 12968_2021_785_MOESM4_ESM.docx]

**ADDITIONAL FILE 4**

**Table. CMR sequence parameters.**

|  | **Cine**  **long-axis** | **Perfusion** | **Cine**  **short-axis** | **TI Scout** | **LGE** |
| --- | --- | --- | --- | --- | --- |
| **Pulse sequence** | bSSFP | Saturation-recovery bSSFP | bSSFP | bSSFP | 3D inversion-recovery fast gradient echo (FGRE) |
| **Field of view (FOV)** | 380 x 340 mm² | 370 x 314 mm² | 380 x 304 mm² | 380 x 307 mm² | 340 x 340 mm² |
| **Slice thickness** | 6 mm | 8 mm | 8 mm | 8 mm | 6 mm |
| **Matrix size** | 256 pixels | 224 pixels | 240 pixels | 208 pixels | 272 pixels |
| **Phase resolution** | 95 % | 80 % | 95 % | 100 % | 89 % |
| **Slice resolution** | NA | NA | NA | NA | 67 % |
| **Phase oversampling** | 0 % | 0 % | 0 % | 0 % | 20 % |
| **Slice oversampling** | NA | NA | NA | NA | 20 % |
| **Voxel size (reconstructed)** | 1.5 x 1.5 x 6 mm^3^ | 1.7 x 1.7 x 8 mm^3^ | 1.6 x 1.6 x 8 mm^3^ | 1.8 x 1.8 x 8 mm^3^ | 1.3 x 1.3 x 6 mm |
| **Voxel size (acquired)** | 1.5 x 1.6 x 6 mm^3^ | 1.7 x 2.0 x 8 mm^3^ | 1.6 x 1.7 x 8 mm^3^ | 1.8 x 1.8 x 8 mm^3^ | 1.3 x 1.4 x 8 mm |
| **TE/TR** | 1.18/2.9 ms | 1.04/2.8 ms | 1.15/2.8 ms | 1.31/3 ms | 1.35/3.4 ms |
| **Flip angle (FA)** | 55° | 70° | 55° | 30° | 9° |
| **Acceleration** | 6.1  (CS) | 2  (GRAPPA) | 6.5  (CS) | 4.2  (CS) | 2  (GRAPPA) |

Abbreviations: bSSFP: balanced steady-state free-precession; CS: compressed-sensing; FGRE: fast gradient echo; FOV: reconstructed field of view; LGE: late gadolinium enhancement; TE: echo time; TI: inversion time; TR: repetition time, NA: not applicable
