# Supplementary material for: Prognostic value of vasodilator stress perfusion cardiovascular magnetic resonance after inconclusive stress testing
Source: J Cardiovasc Magn Reson. 2021 Jul 5;23:89. doi: 10.1186/s12968-021-00785-6 (PMC8256486; doi:10.1186/s12968-021-00785-6)
Supplement: Supplementary file 5 — Additional file 5. Safety results. [file 12968_2021_785_MOESM5_ESM.docx]

**ADDITIONAL FILE 5**

**Safety results**

There were one case of unstable angina and two acute pulmonary edemas, but no transient ischemic attack, disabling stroke, ST elevation MI or sustained ventricular tachycardia were recorded in relation to stress CMR. Among the 1,563 patients who completed the stress CMR protocol, the main adverse events during or immediately after the study were as follows: 287 headaches (18.4%), 153 chest discomfort due to dipyridamole (9.8%), 124 nausea or vomiting (7.9%), 45 dizziness (2.9%) and 31 anginas with ECG evidence of myocardial ischemia (2.0%). For all patients, symptoms resolved quickly with intravenous theophylline and additional sublingual nitrates and/or intravenous betablockers in 17 patients (1.1%).
