# Supplementary material for: Prognostic value of vasodilator stress perfusion cardiovascular magnetic resonance after inconclusive stress testing
Source: J Cardiovasc Magn Reson. 2021 Jul 5;23:89. doi: 10.1186/s12968-021-00785-6 (PMC8256486; doi:10.1186/s12968-021-00785-6)
Supplement: Supplementary file 6 — Additional file 6. Table. Baseline characteristics of patients according to the presence of symptoms. [file 12968_2021_785_MOESM6_ESM.docx]

**ADDITIONAL FILE 6**

**Table. Baseline characteristics of patients according to the presence of symptoms (N=1,402).**

|  | **All patients**  **(N=1,402)** | **Patients**  **without**  **symptom**  **(N=485)** | **Patients**  **with**  **symptom**  **(N=917)** | **p value** |
| --- | --- | --- | --- | --- |
|  |  |  |  |  |
| Age, years | 69.5 ± 11.0 | 72.2 ± 11.0 | 68.1 ± 10.9 | **<0.001** |
| Males, n (%) | 935 (66.7) | 368 (75.9) | 567 (61.8) | **<0.001** |
| Body mass index, kg/m² | 28.6 ± 6.3 | 28.5 ± 7.2 | 28.6 ± 6.2 | 0.162 |
|  |  |  |  |  |
| Coronary risk factors, n (%) |  |  |  |  |
| Diabetes mellitus | 459 (32.7) | 201 (41.4) | 258 (28.1) | **<0.001** |
| Hypertension | 807 (57.6) | 285 (58.8) | 522 (56.9) | **0.021** |
| Obesity^*^ | 431 (30.7) | 148 (30.5) | 283 (30.9) | 0.451 |
| Dyslipidemia | 819 (58.4) | 286 (59.0) | 533 (58.1) | 0.128 |
| Smoking | 336 (24.0) | 151 (31.1) | 185 (20.2) | **<0.001** |
| Family history of CAD | 391 (27.9) | 132 (27.2) | 259 (28.2) | 0.092 |
|  |  |  |  |  |
| Medical history of CVD, n (%) |  |  |  |  |
| Known CAD | 727 (51.9) | 422 (87.0) | 305 (33.3) | **<0.001** |
| - History of PCI | 442 (31.5) | 309 (63.7) | 133 (14.5) | **<0.001** |
| - History of CABG | 445 (31.7) | 179 (36.9) | 266 (29.0) | **<0.001** |
| - Known MI | 340 (24.3) | 198 (40.8) | 142 (15.5) | **<0.001** |
| Peripheral atheroma | 158 (11.3) | 97 (20.0) | 61 (6.7) | **<0.001** |
| Ischemic stroke | 54 (3.9) | 19 (3.9) | 35 (3.8) | 0.691 |
|  |  |  |  |  |
| ESC SCORE – Ten-year risk for fatal CAD^†^, % | 2.5 (1.2-5.9) | 3.4 (2.2-6.9) | 2.1 (0.7-5.5) | **<0.001** |

*Values are n (%), mean ± SD, or median (interquartile range).*

* defined by BMI ≥30 kg/m2

^†^ based on a modified ESC SCORE project (https://www.escardio.org/Education/Practice-Tools/CVD-prevention-toolbox/SCORE-Risk-Charts) that did not take into account the total cholesterol level. (22-23)

*Abbreviations: BMI: body mass index; CABG: coronary artery bypass graft; CAD: coronary artery disease; CVD: cardiovascular disease; ECG: electrocardiogram; HF: heart failure; MI: myocardial infarction; PCI: percutaneous coronary intervention.*
