# Supplementary material for: Prognostic value of vasodilator stress perfusion cardiovascular magnetic resonance after inconclusive stress testing
Source: J Cardiovasc Magn Reson. 2021 Jul 5;23:89. doi: 10.1186/s12968-021-00785-6 (PMC8256486; doi:10.1186/s12968-021-00785-6)
Supplement: Supplementary file 7 — Additional file 7. Figure. Examples of inducible myocardial ischemia on stress CMR in patients with a prior inconclusive stress test. [file 12968_2021_785_MOESM7_ESM.docx]

**ADDITIONAL FILE 7**

**Figure. Examples of inducible myocardial ischemia on stress CMR in patients with a prior inconclusive stress test.**

**
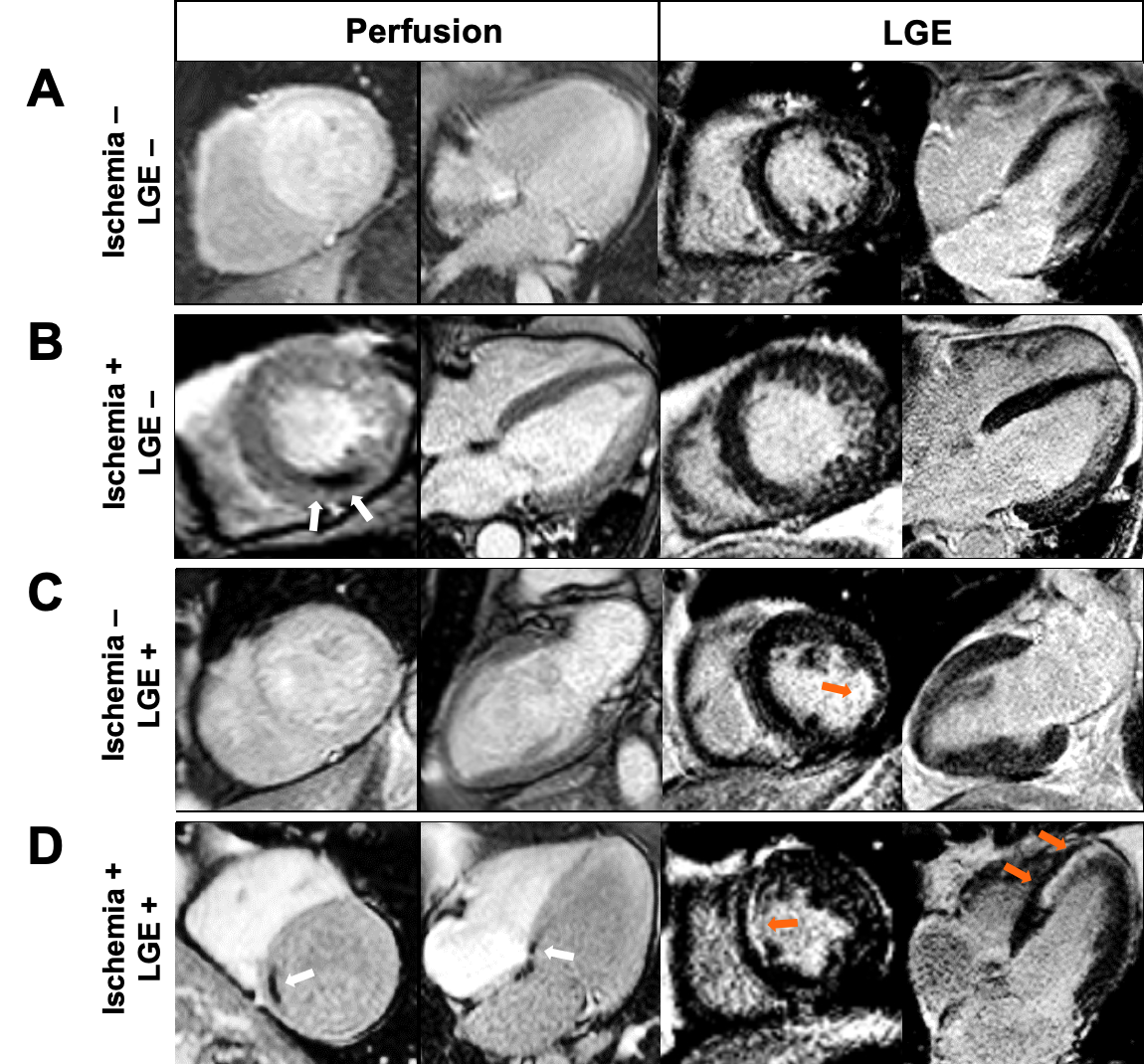
**

**Panel A: normal.** 79-year-old female with a prior inconclusive exercise ECG, presenting atypical angina. Stress CMR revealed no perfusion defect and LGE was negative, ruling out the diagnosis of myocardial ischemia.

**Panel B: inducible ischemia.** 81-year-old male with a prior inconclusive SPECT, presenting dyspnea on exertion. First-pass myocardial stress perfusion images revealed a reversible perfusion defect of the inferior wall (*white arrows*) without LGE, indicative of myocardial inducible ischemia suggestive of significant right coronary artery (RCA) stenosis, confirmed by coronary angiography.

**Panel C: myocardial scar without ischemia.** 75-year-old female with a history of lateral ST elevation MI (STEMI) treated by percutaneous coronary intervention (PCI) of the left circumflex coronary artery (LCX) 6 years previously and a prior inconclusive stress echocardiography, presenting atypical angina. Stress CMR showed a subendocardial lateral scar on LGE (*orange arrow*), without any perfusion defect and, therefore, no inducible ischemia.

**Panel D: myocardial scar with additional inducible ischemia.** 68-year-old male with a history of anterior STEMI treated by PCI of the left anterior descending coronary artery (LAD) and with a prior inconclusive stress echocardiography, presenting dyspnea on exertion. Stress CMR showed a subendocardial scar on the antero-septo-apical wall on LGE sequences (*orange arrows*), and a perfusion defect of the inferior and infero-septal wall (*white arrows*) on first-pass perfusion images, indicative of inducible myocardial ischemia. Coronary angiography revealed high-grade stenoses of one branch of the RCA.

*Abbreviations: CAD: coronary artery disease; CMR: cardiovascular magnetic resonance; Cx: circumflex coronary artery; ECG: electrocardiogram; LAD: left anterior descending; LGE: late gadolinium enhancement; MI: myocardial infarction; NSTEMI:* *non-ST segment elevation myocardial infarction; PCI: percutaneous coronary intervention; RCA: right coronary artery;* *SPECT: single photon emission computed tomography; STEMI: ST segment elevation myocardial infarction.*
