# Supplementary material for: Prognostic value of vasodilator stress perfusion cardiovascular magnetic resonance after inconclusive stress testing
Source: J Cardiovasc Magn Reson. 2021 Jul 5;23:89. doi: 10.1186/s12968-021-00785-6 (PMC8256486; doi:10.1186/s12968-021-00785-6)
Supplement: Supplementary file 8 — Additional file 8. Figure. Annualized rates of MACE stratified by the presence of myocardial ischemia and late gadolinium enhancement (LGE). [file 12968_2021_785_MOESM8_ESM.docx]

**ADDITIONAL FILE 8**

**Figure. Annualized rates of MACE stratified by the presence of myocardial ischemia and late gadolinium enhancement (LGE).**

Annual event rates of MACE (cardiovascular mortality and nonfatal MI) for the entire study cohort. Patients without inducible ischemia or LGE had a lower annualized rate of MACE (2.1%/year) than patients with inducible ischemia without or with LGE (9.0%/year and 9.3%/year, respectively; both p<0.001).

**
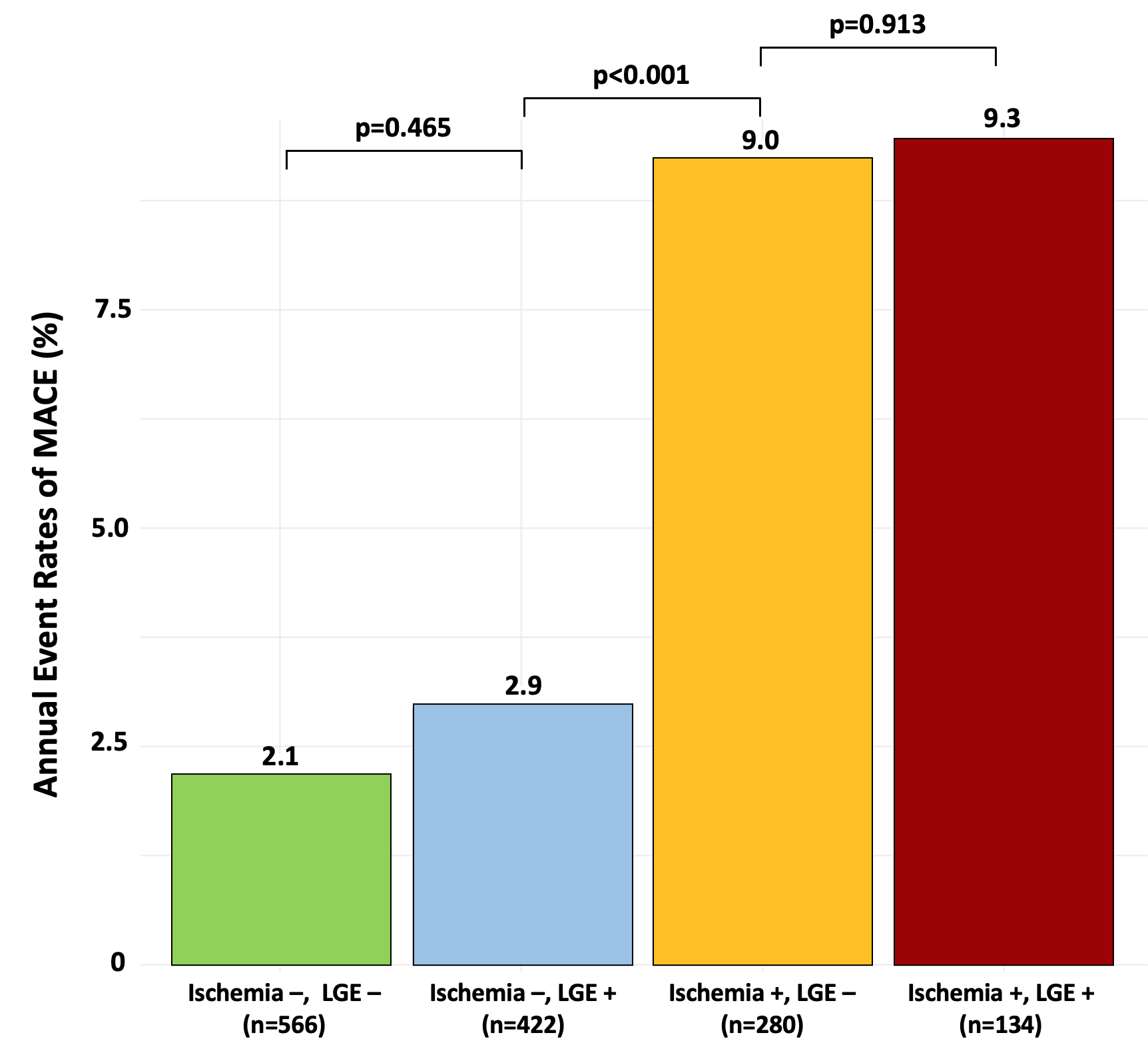
**
