# Supplementary material for: Prognostic value of vasodilator stress perfusion cardiovascular magnetic resonance after inconclusive stress testing
Source: J Cardiovasc Magn Reson. 2021 Jul 5;23:89. doi: 10.1186/s12968-021-00785-6 (PMC8256486; doi:10.1186/s12968-021-00785-6)
Supplement: Supplementary file 9 — Additional file 9. Figure. Age interaction: annualized event rates of MACE stratified by age and presence/absence of myocardial ischemia. [file 12968_2021_785_MOESM9_ESM.docx]

**ADDITIONAL FILE 9**

**Figure. Age interaction: annualized event rates of MACE stratified by age and presence/absence of myocardial ischemia.**

Annualized event rates of MACE are stratified by presence/absence of myocardial ischemia in different age categories: < 50 years; 50-60 years; 60-70 years and > 70 years.

**
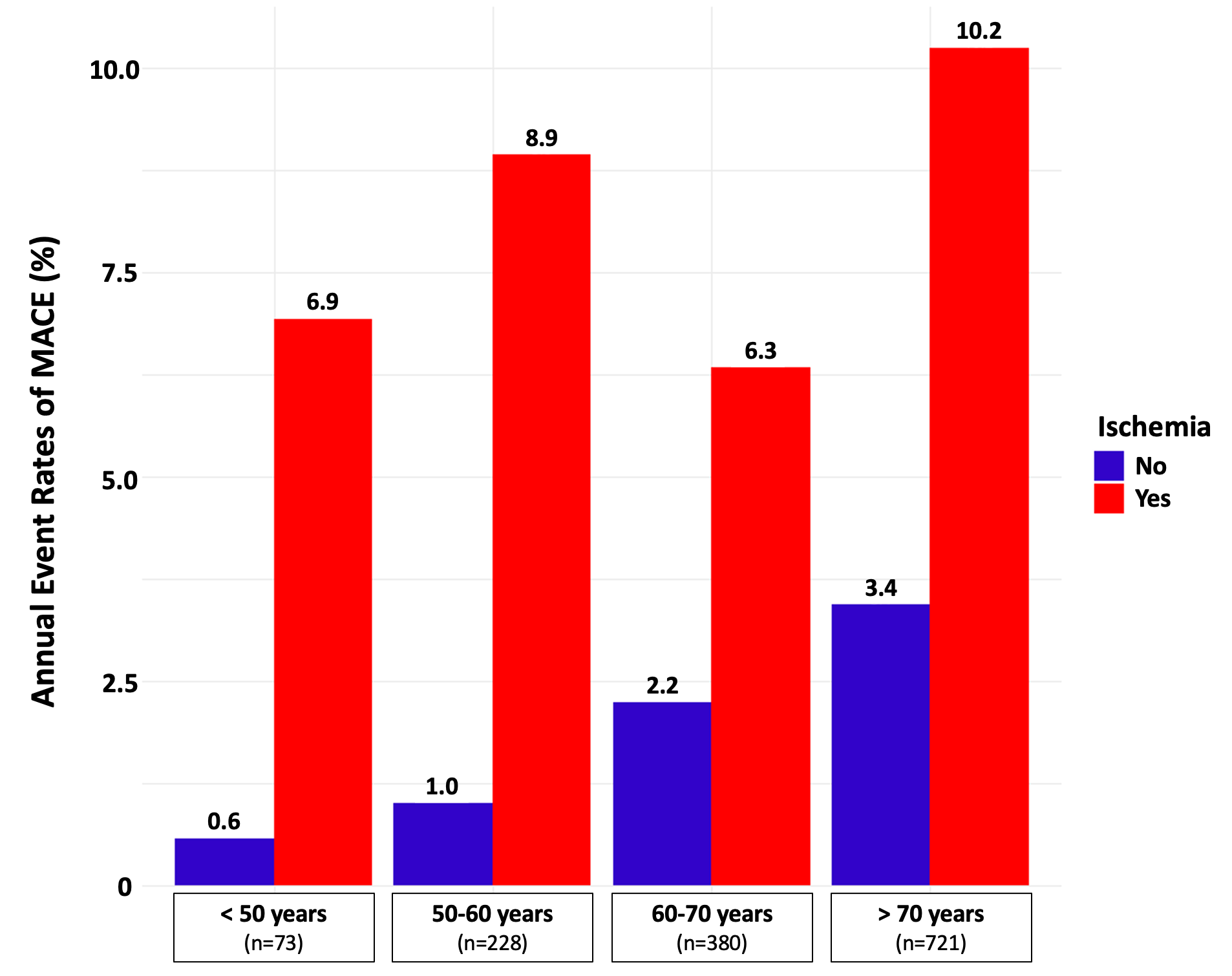
**
