# Supplementary material for: Prognostic value of vasodilator stress perfusion cardiovascular magnetic resonance after inconclusive stress testing
Source: J Cardiovasc Magn Reson. 2021 Jul 5;23:89. doi: 10.1186/s12968-021-00785-6 (PMC8256486; doi:10.1186/s12968-021-00785-6)
Supplement: Supplementary file 10 — Additional file 10. Figure. Kaplan-Meier Curves for MACE stratified by the presence of ischemia and according to patient sex. [file 12968_2021_785_MOESM10_ESM.docx]

**ADDITIONAL FILE 10**

**Figure. Kaplan-Meier Curves for MACE stratified by the presence of ischemia and according to patient sex.**

Kaplan Meier curves for MACE (cardiovascular mortality or nonfatal MI) as a function of length of follow-up for women (A) or men (B), stratified by presence or absence of inducible ischemia. Test comparing the two groups was based on the log-rank test.

**
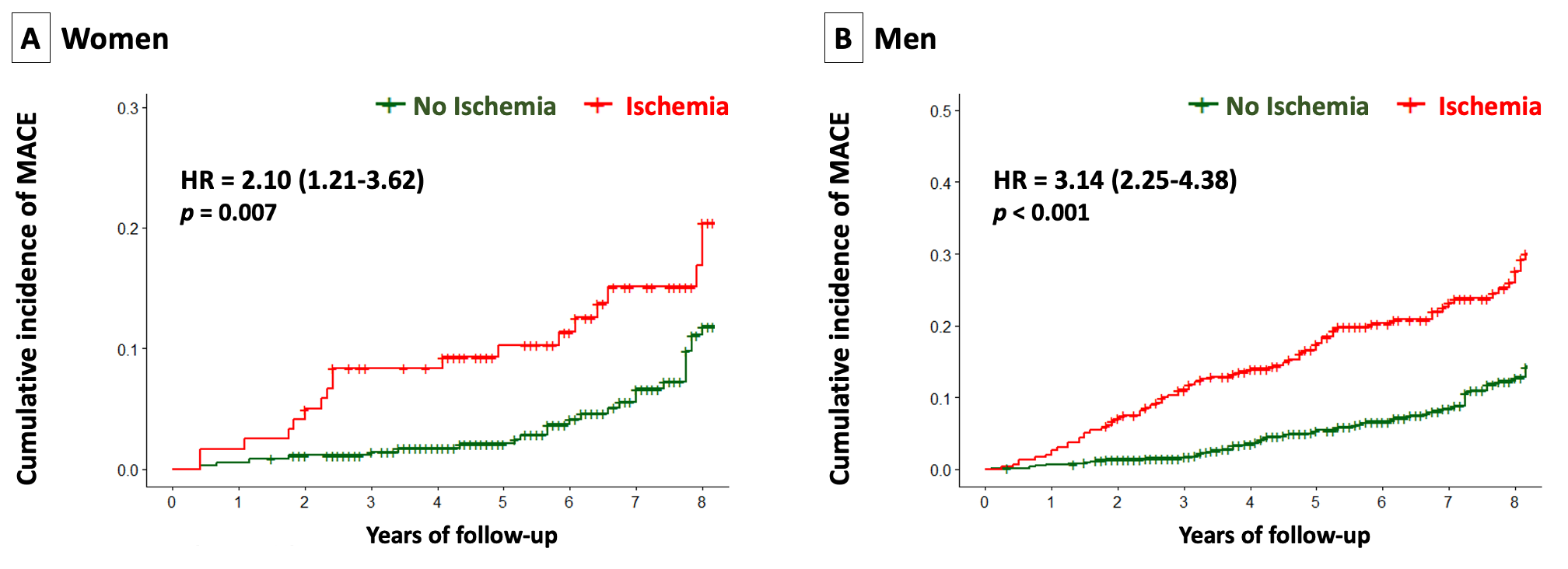
**
