# Supplementary material for: Prognostic value of vasodilator stress perfusion cardiovascular magnetic resonance after inconclusive stress testing
Source: J Cardiovasc Magn Reson. 2021 Jul 5;23:89. doi: 10.1186/s12968-021-00785-6 (PMC8256486; doi:10.1186/s12968-021-00785-6)
Supplement: Supplementary file 12 — Additional file 12. Table. Univariable and multivariable analysis of CMR-induced myocardial ischemia for prediction of adverse events. [file 12968_2021_785_MOESM12_ESM.docx]

**ADDITIONAL FILE 12**

**Table. Univariable and multivariable analysis of CMR-induced myocardial ischemia for prediction of adverse events.**

|  | **Univariable analysis** | |  | **Multivariable analysis*** | |
| --- | --- | --- | --- | --- | --- |
|  | **Hazard Ratio**  **(95% CI)** | **p value** |  | **Hazard Ratio**  **(95% CI)** | **p value** |
|  |  |  |  |  |  |
| ***Primary outcome (MACE)*** |  |  |  |  |  |
| Cardiovascular mortality | 2.44 (1.75–3.40) | **<0.001** |  | 1.83 (1.29–2.60) | **<0.001** |
| Nonfatal MI | 5.04 (3.09–8.21) | **<0.001** |  | 5.14 (3.11–8.52) | **<0.001** |
|  |  |  |  |  |  |
| ***Secondary outcomes*** |  |  |  |  |  |
| All-cause of mortality | 1.73 (1.35–2.22) | **<0.001** |  | 1.24 (0.96–1.61) | 0.103 |
| Late coronary revascularization without emergency | 2.61 (1.76–3.87) | **<0.001** |  | 2.46 (1.64–3.69) | **<0.001** |
| Hospitalization for heart failure | 0.94 (0.61–1.46) | 0.801 |  | 0.85 (0.54–1.34) | 0.495 |
| Ventricular arrythmias | 2.76 (1.41–5.42) | **0.003** |  | 2.92 (1.44–5.90) | **0.003** |
|  |  |  |  |  |  |

* Covariates in the model by stepwise variable selection with entry and exit criteria set at the p≤0.1 level for MACE (model 2a): age, male, hypertension, diabetes, dyslipidemia, known CAD, LVEF per 10%, the presence of LGE and the presence of ischemia.

*Abbreviations*: CAD: coronary artery disease; CI: confidence interval; CMR: cardiovascular magnetic resonance; HR: hazard ratio; LGE: late gadolinium enhancement; LVEF: left ventricular ejection fraction; MACE: major adverse cardiac events; MI: myocardial infarction.
