# Supplementary material for: Prognostic value of vasodilator stress perfusion cardiovascular magnetic resonance after inconclusive stress testing
Source: J Cardiovasc Magn Reson. 2021 Jul 5;23:89. doi: 10.1186/s12968-021-00785-6 (PMC8256486; doi:10.1186/s12968-021-00785-6)
Supplement: Supplementary file 13 — Additional file 13. Table. Univariable and Multivariable Competing Risk Regression Analysis. [file 12968_2021_785_MOESM13_ESM.docx]

**ADDITIONAL FILE 13**

**Table. Univariable and Multivariable Competing Risk Regression Analysis.**

| **Nonfatal MI Cardiovascular Mortality** | | | | | | | | | | |  |
| --- | --- | --- | --- | --- | --- | --- | --- | --- | --- | --- | --- |
|  |  |  |  |  |  |  |  |  |  |  |  |
|  | **Univariable analysis** | |  | **Multivariable analysis^*^** | |  | **Univariable analysis** | |  | **Multivariable analysis^*^** | |
|  | **sHR^†^ (95% CI)** | **p value** |  | **sHR^†^ (95% CI)** | **p value** |  | **sHR^†^ (95% CI)** | **p value** |  | **sHR^†^ (95% CI)** | **p value** |
|  |  |  |  |  |  |  |  |  |  |  |  |
| Age | 1.02 (0.99–1.04) | 0.15 |  | - | - |  | 1.05 (1.03–1.07) | **<0.001** |  | 1.06 (1.03–1.08) | **<0.001** |
| Male | 1.30 (0.79–2.15) | 0.31 |  | - | - |  | 1.74 (1.17–2.60) | **0.006** |  | 1.47 (0.97–2.23) | 0.07 |
| BMI | 1.01 (0.98–1.05) | 0.37 |  | - | - |  | 1.05 (0.97–1.06) | 0.59 |  | - | - |
| Hypertension | 1.18 (1.01–1.54) | **0.04** |  | - | - |  | 1.30 (0.90–1.87) | 0.16 |  | - | - |
| Diabetes mellitus | 1.01 (0.88-1.33) | 0.45 |  | - | - |  | 1.66 (1.17–2.37) | **<0.001** |  | 1.57 (1.06–2.35) | **0.026** |
| Dyslipidemia | 1.20 (0.75–1.92) | 0.44 |  | - | - |  | 1.55 (1.07–2.25) | **0.02** |  | 1.34 (0.91–1.97) | 0.14 |
| Smoking | 1.07 (0.82–1.40) | 0.54 |  | - | - |  | 1.01 (0.79–1.31) | 0.67 |  | - | - |
| Family history of CAD | 0.73 (0.43–1.25) | 0.26 |  | - | - |  | 1.19 (0.82–1.73) | 0.36 |  | - | - |
| Known CAD | 1.27 (0.80–2.01) | 0.31 |  | - | - |  | 1.33 (0.94–1.89) | 0.09 |  | 1.73 (1.06–2.82) | **0.028** |
| Known MI | 0.83 (0.45–1.50) | 0.53 |  | - | - |  | 0.87 (0.55–1.37) | 0.54 |  | - | - |
| Peripheral atheroma | 1.53 (0.74–3.15) | 0.25 |  | - | - |  | 1.20 (0.65–2.21) | 0.57 |  | - | - |
| Ischemic stroke | 1.27 (0.46–3.51) | 0.65 |  | - | - |  | 0.83 (0.35–1.94) | 0.67 |  | - | - |
| History of hospitalization for HF | 1.20 (0.38–3.76) | 0.75 |  | - | - |  | 1.43 (0.62–3.26) | 0.40 |  | - | - |
| Presence of inducible ischemia | 4.26 (2.60–6.89) | **<0.001** |  | 4.74 (2.92–7.68) | **<0.001** |  | 1.80 (1.27–2.56) | **<0.001** |  | 1.77 (1.25–2.51) | **<0.001** |
| Number of ischemic segments | 1.61 (1.44–1.79) | **<0.001** |  | 1.63 (1.48–1.82) | **<0.001** |  | 1.40 (1.28–1.53) | **<0.001** |  | 1.22 (1.08–1.40) | **<0.001** |
| Presence of LGE | 1.52 (1.18–2.29) | **0.02** |  | - | - |  | 1.41 (1.04–2.03) | **0.042** |  | - | - |
| Number of segments of LGE | 1.29 (1.14–1.47) | **<0.001** |  | 1.27 (1.12–1.45) | **<0.001** |  | 1.31 (1.19–1.45) | **<0.001** |  | 1.23 (1.10–1.38) | **<0.001** |
| LVEF^‡^ | 0.99 (0.97–1.00) | 0.13 |  | - | - |  | 0.84 (0.76-0.96) | **<0.001** |  | 0.92 (0.79–1.05) | 0.22 |
| LV end-diastolic volume index^‡^ | 1.08 (1.05-1.10) | **0.019** |  | 1.03 (1.01–1.07) | **0.03** |  | 1.02 (0.96–1.08) | 0.87 |  | - | - |
| LV end-systolic volume index^‡^ | 1.10 (1.07-1.13) | **0.043** |  | - | - |  | 1.03 (0.98–1.09) | 0.69 |  | - | - |
|  |  |  |  |  |  |  |  |  |  |  |  |

* Covariates in the **model by stepwise variable selection** with entry and exit criteria set at the p≤0.1 level:

- for nonfatal MI: hypertension, LVEF per 10 %, number segments of LGE, CMR-related coronary revascularization and the presence of inducible ischemia or number of ischemic segments.
- for CV mortality: age, male, diabetes, dyslipidemia, known CAD, LVEF per 10 %, number segments of LGE, CMR-related coronary revascularization and the presence of inducible ischemia or number of ischemic segments.

Of note, the presence of inducible ischemia and the number of ischemic segments were not included simultaneously in these models, but are each calculated in their respective models to be investigated individually.

^†^ HR of the subdistribution hazard function

^‡^ Increment of 10 units.

*Abbreviations*: CAD: coronary artery disease; CI: confidence interval; CMR: cardiovascular magnetic resonance; HR: hazard ratio; LGE: late gadolinium enhancement; LVEF: left ventricular ejection fraction; MACE: major adverse cardiac events; MI: myocardial
